# Expression of WASF3 in patients with non-small cell lung cancer: Correlation with clinicopathological features and prognosis

**DOI:** 10.3892/ol.2014.2276

**Published:** 2014-06-23

**Authors:** JIE WU, GUANG-CHUAN WANG, XUE-JUN CHEN, ZHAN-RUI XUE

**Affiliations:** 1 Department of Oncology, The First Affiliated Hospital of Liaoning Medical University, Liaoning, Jinzhou 121000, P.R. China; 2 Department of Immunology, Liaoning Medical University, Liaoning, Jinzhou 121000, P.R. China; 3 Department of Pathology, Liaoning Medical University, Liaoning, Jinzhou 121000, P.R. China

**Keywords:** non-small cell lung cancer, survival analysis, prognosis, Wiskott-Aldrich syndrome protein family member 3

## Abstract

Wiskott-Aldrich syndrome protein family member 3 (WASF3) is required for invasion and metastasis in different cancer cell types, and has been demonstrated to possess prognostic value in various types of human cancer. However, to the best of our knowledge, the expression profile of WASF3 and its correlations with the clinicopathological features of non-small cell lung cancer (NSCLC) have not yet been described. In the present study, the mRNA expression levels of WASF3, in 38 NSCLC patients and in matched normal tissues, were assessed using quantitative polymerase chain reaction and the protein expression in 96 specimens was analyzed using immunohistochemistry. In addition, patient survival data were collected retrospectively and the association between WASF3 expression and five-year overall survival was evaluated. The results demonstrated that the mRNA expression level of WASF3 in cancer tissues was markedly (approximately five times) higher compared with that of the normal tissues. The WASF3 protein expression profile in NSCLC was consistent with the mRNA expression result, which also correlated with the histological subtype and tumor stage. Furthermore, patients with WASF3-positive expression were associated with a poorer prognosis compared with those exhibiting WASF3-negative expression, and the five-year survival rate was 20.8 and 46.5%, respectively (Kaplan-Meier; log-rank, P=0.004). In the multivariate analysis, which included other clinicopathological features, WASF3 emerged as an independent prognostic factor (relative risk, 0.463; 95% CI, 0.271–0.792). These results indicate that WASF3 may be critical in the pathogenesis of NSCLC, in addition to being a valuable prognostic factor for NSCLC patients. Further investigations are required to identify the efficacy of WASF3 as a potential therapeutic target for the treatment of NSCLC.

## Introduction

Lung cancer remains the leading cause of cancer mortalities worldwide, particularly, in economically developed countries, although marginal progress has been made in the treatment of lung cancer (1). Based on its histologic features and responses to conventional therapeutic strategies, lung cancer has been divided into two major categories: Non-small cell lung cancer (NSCLC; one of the most lethal types of cancer) and small cell lung cancer. Due to the incurable nature of lung cancer, NSCLC is considered to be a terminal illness with a five-year survival rate of ~16% (2). The poor prognosis is predominantly attributed to metastasis in the early stages of NSCLC. Therefore, investigating the potential biological markers of cancer invasion and metastasis is urgently required for guidance on postoperative surveillance and therapeutic decisions.

Cancer invasion and metastasis is a multistep process, which involves tumor cells escaping from a primary site, migrating into the blood or lymphatic system and re-establishing novel tumors at a distant site. Various signaling molecules that regulate multiple cellular processes are involved in cell migration (3). For example, the actin cytoskeleton is dynamically remodeled during cell migration, and this reorganization produces the force that is necessary for cell migration (4). Previous studies identified that Wiskott-Aldrich syndrome protein family 3 (WASF3; also termed, WAVE3), is critical for the regulation of actin cytoskeleton dynamics via the activation of the Arp2/3 complex, and is involved in cancer cell motility, invasion and metastasis (5–8). Downregulation of WASF3 has been found to inhibit the invasion and metastasis of breast cancer cells, and has been proposed as a metastasis promoter gene (8). In addition, it has been reported that, compared with lower stage tumors and normal tissue, WASF3 expression is increased in advanced breast and prostate cancer (9,10). However, currently, little is known about the expression status of WASF3 and its association with the clinicopathological features of NSCLC. In the present study, the mRNA expression levels of WASF3 were analyzed in 38 NSCLC patients and in matched normal tissues, and the protein expression status in 96 specimens was analyzed using the quantitative polymerase chain reaction (qPCR) and immunohistochemistry IHC. The correlations between WASF3 expression patterns and the clinicopathological features of NSCLC were analyzed. In addition, the association between WASF3 expression and the five-year overall survival was evaluated.

## Patients and methods

### Study population and samples

138 NSCLC patients who were diagnosed with, and underwent surgical removal of, a primary lesion at the First Affiliated Hospital of Liaoning Medical University (Liaoning, China) were included in the present study. None of the patients underwent radiotherapy or chemotherapy prior to surgery. Histopathological evaluation was conducted independently by two pathologists. All of the clinical and follow-up data were based on studies from the tumor registry office of the First Affiliated Hospital of Liaoning Medical University. The present study was approved by the Ethics Committee of Liaoning Medical University and written informed consent was acquired from each patient. One-hundred lung cancer tissue samples from these patients were formalin-fixed, paraffin-embedded and prepared for IHC. Postoperative follow-up endured for at least five years for 96 patients, while four patients failed to be followed up and were excluded from the study. Fresh tumor tissues and matched normal tissues from an additional 38 NSCLC patients were immediately transferred to liquid nitrogen and stored at −80°C for subsequent qPCR. Clinicopathological data are summarized in Table I.

### Total RNA extraction and cDNA synthesis

Total RNA was extracted from the tissues using the TRIzol RNA kit (Invitrogen Life Technologies, Carlsbad, CA, USA) in accordance with the manufacturer’s instructions. Ultraviolet spectroscopy was performed using an Eppendorf BioPhotometer (Eppendorf, Hamburg, Germany) to determine the RNA concentration and purity. Total RNA (2 μg) was reverse-transcribed into first-strand cDNA using the first-strand PrimeScript™ RT Reagent kit with gDNA Eraser (Takara Bio, Inc., Japan), according to the manufacturer’s instructions. The final reaction volume was 20 μl.

### qPCR

qPCR using the SYBR^®^ Green I (Takara Bio, Inc.) technique was adopted to examine the WASF3 expression levels of the tissues from the 38 NSCLC patients. Primers spanning at least one intron were chosen to minimize inaccuracies due to genomic DNA contamination. The housekeeping gene, glyceraldehydes-3-phosphate dehydrogenase (GAPDH) served as an internal control. The primer sequences used were as follows: Sense, 5′-TGA TAA CTG AGC CAA AGT GGT GAT G-3′ and antisense, 5′-TGG CGT ATG ATA GCG GCA AG-3′ (PCR product length, 198 bp) for WASF3; and sense, 5′-GCA CCG TCA AGG CTG AGA AC-3′ and antisense, 5′-TGG TGA AGA CGC CAG TGG A-3′ (PCR product length, 138 bp) for GAPDH. The qPCR was run on a Mastercycler^®^ ep realplex (Eppendorf) using a SYBR^®^ Premix Ex Taq™ kit (Takara Bio, Inc.). Briefly, PCR (total volume, 20 μl) was performed with 2 μl cDNA, 0.2 μM each primer pair and 10 μl SYBR^®^ Premix Ex Taq (2× concentration). Following the initial denaturation at 95°C for 30 sec, 40 three-segment cycles consisting of the following procedure were performed: 5 sec at 95°C; 30 sec at 55°C; and 30 sec at 72°C. The fluorescence was automatically measured using the Mastercycler^®^ ep realplex (Eppendorf) during PCR and during one three-segment cycle of product melting (95°C for 15 sec, 60°C for 15 sec, 95°C for 15 sec). In order to further verify the amplification of the desired fragments, the PCR products were assessed via electrophoresis analysis on 3% agarose gel. The 2^−ΔΔCT^ method was used to present the data of the genes of interest relative to an internal control gene (11,12).

### IHC

Paraffin-embedded 4-μm thick tissue sections were deparaffinized in a series of xylene baths and rehydrated during graded alcohol washes. All sections were retrieved by microwave treatment and treated with 3% hydrogen peroxidase for 15 min to block endogenous peroxidase activity. The sections were subsequently incubated with the primary anti-WASF3 rabbit antibody (1:100; ab-110739; Abcam, Cambridge, UK) at 4°C overnight. Thereafter, the sections were stained with a ready-to-use secondary anti-rabbit antibody conjugated with horseradish peroxidase (Zhongshan Biotechnology Inc., Beijing, China) for 30 min at room temperature. The stained specimens were exposed to a 3,3′-diaminobenzidine kit (Zhongshan Biotechnology Inc., Beijing, China) at room temperature for 1 min and counterstained with hematoxylin. The primary antibodies were replaced with phosphate-buffered saline to serve as the negative controls. All slides were independently examined and scored by two pathologists, who were blind to the clinical and pathological data of the subjects. Cancer cells in at least five fields were counted at a magnification of ×200 using a Zeiss Imager A1 (Carl Zeriss AG, Oberkochen, Germany). For the WASF3 IHC assessment, the ratio of positive cells per specimen and staining intensity were analyzed. The WASF3 immunoreactivity level was classified by the proportion of positive cells as follows: 0, <5% positive cells; 1+, 5–30% positive cells; 2+, >30–50% positive cells; and 3+, >50% positive cells. In addition, the intensity of WASF3 expression was scored: 0, Negative to weak; 1, moderate; and 2, strong. The score was the sum of the intensity and the percentage of positive cells. A score of ≤1 was applied as a cut-off point for loss of WASF3 expression.

### Statistical analysis

A two-sample t-test for independent samples was used for the continuous variables. Statistical analysis for comparing between groups regarding categorical data was performed using the χ^2^-test. Comparison of more than two groups with continuous variables was performed with the Kruskal-Wallis one-way analysis of variance by ranks. The Kaplan-Meier method with log-rank test was used for comparing survival curves between the groups. Cox regression (or proportional hazards regression) was adopted to analyze the effect of various risk factors on survival. SPSS software version 16.0 (SPSS Inc., Chicago, IL, USA) was used for statistical analysis, tests were two-sided and P<0.05 was considered to indicate a statistically significant difference.

## Results

### mRNA expression of WASF3 in NSCLC and correlation with clinicopathological features of NSCLC

**Figure 1:**
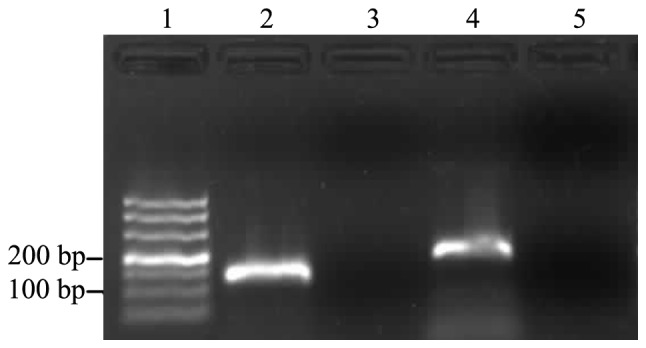
Gel electrophoresis analysis of the quantitative polymerase chain reaction products. Lanes: 1, 500 bp molecular size marker; 2, glyceraldehydes-3-phosphate dehydrogenase (GAPDH); 3, no template control for GAPDH; 4, Wiskott-Aldrich syndrome protein family member 3 (WASF3); and 5, no template control for WASF3.

**Figure 2:**
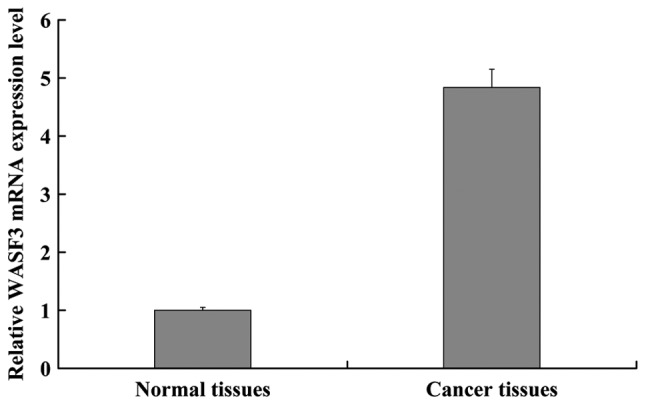
mRNA expression levels of WASF3 in normal tissues and non-small cell lung cancer tissues. WASF3, Wiskott-Aldrich syndrome protein family member 3.

The mRNA expression levels of WASF3 in 38 NSCLC patients and in matched normal lung tissue samples were quantitatively assessed using qPCR and the SYBR^®^ Green I technique. Gel electrophoresis analysis of the amplification products revealed a single band with the anticipated size for WASF3 (198 bp) and GAPDH (138 bp; Fig. 1). Furthermore, melting curve analysis identified the specific amplification of the target and reference genes. The mRNA expression level of WASF3 was markedly (approximately five times) higher in the NSCLC tissues (4.8373±0.3142) compared with that in the normal tissues (1.000) (Fig. 2).

The association between WASF3 mRNA expression in the NSCLC tissues and various clinicopathological features was analyzed (Table II). The expression of WASF3 was significantly higher in adenocarcinoma compared with that in squamous cell carcinoma (P=0.010). There was a significant correlation observed between WASF3 expression and the tumor stage (P=0.013). However, significant correlation between WASF3 mRNA expression, and lymph node metastasis status and differentiation status (P=0.815 and P=0.214, respectively) was not detected.

### Expression of the WASF3 protein in NSCLC and correlation with clinicopathological features

**Figure 3:**
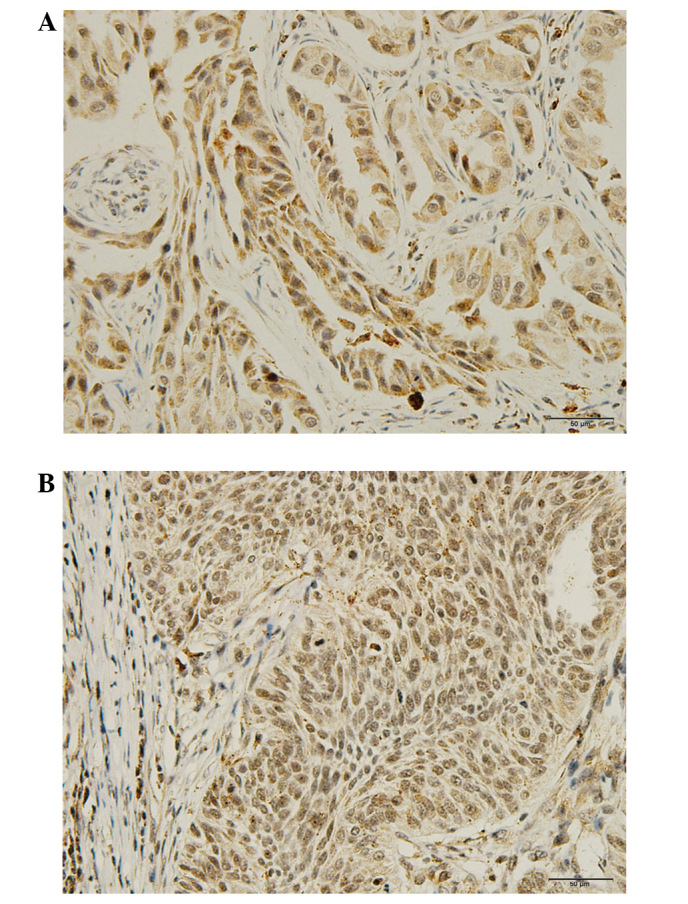
Immunostaining of Wiskott-Aldrich syndrome protein family member 3 in non-small cell lung cancer samples; (A) adenocarcinoma and (B) squamous cell carcinoma (magnification, ×400).

IHC demonstrated that the WASF3 protein was predominantly localized to the cytoplasm (Fig. 3). Positive WASF3 expression was observed in 53 (55.2%) cases and negative expression was noted in 43 (44.8%) cases out of the 96 patients. As anticipated, it was found that WASF3 expression in the NSCLC cases was significantly correlated with the histological subtype and tumor staging (P=0.001 and P=0.024, respectively), however, was not correlated with gender, age, differentiation status or lymph node metastasis. Detailed results are presented in Table III.

### Correlation between expression of WASF3 and overall survival

**Figure 4:**
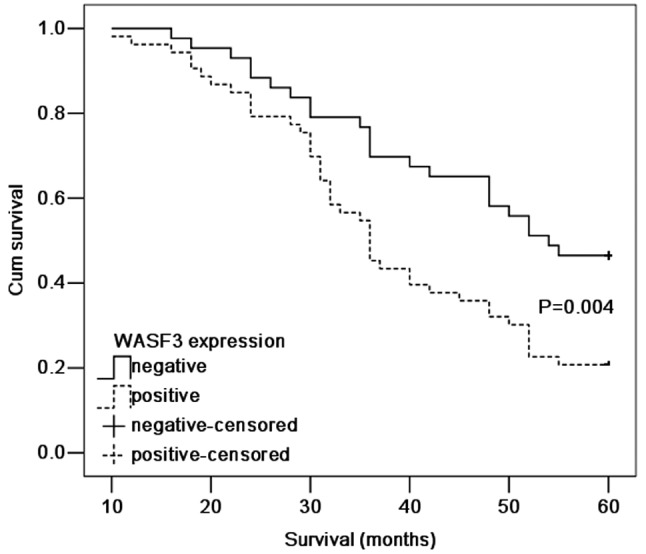
Kaplan-Meier curves showing five-year overall survival of non-small cell lung cancer patients with regard to WASF3 expression. Log-rank test, P=0.004. WASF3, Wiskott-Aldrich syndrome protein family member 3.

According to the Kaplan-Meier survival analysis, patients exhibiting WASF3-positive expression had a poorer prognosis (P=0.004; Fig. 4) compared with those patients with WASF3-negative expression. The five-year survival rate for patients with high expression of WASF3 was 20.8% compared with 46.5% in patients exhibiting a low expression. The Cox proportional hazards model was adopted to perform univariate and multivariate analysis of survival. As a result of the univariate analysis, the tumor staging (P=0.005) and WASF3 expression (P=0.006) were identified to be associated with overall survival. In the multivariate analysis, the expression of WASF3 emerged as an independent and significant factor, which was associated with a poor five-year survival rate (relative risk, 0.463; 95% CI, 0.271–0.792). Detailed results are presented in Table IV.

## Discussion

Metastasis is accountable for ~90% of mortalities in patients with solid tumors (13–18) and is the most problematic issue during cancer treatment. Mechanistic and clinical studies have clearly demonstrated WASF3 as a critical component in cancer progression and metastasis (19). Furthermore, recent studies have reported on the critical role of WASF3 in numerous malignancies, including prostate (9,20), breast (21) and colon cancer (22). However, there is limited data available regarding the expression status of WASF3 in NSCLC. In the present study, the results of IHC and qPCR clearly demonstrate that WASF3 expression was increased in the tumor tissue samples, which indicates a potential role for the WASF3 protein in the pathogenesis of NSCLC. In addition, the present data demonstrated that WASF3 expression was significantly correlated with the histological subtype and tumor staging, which is consistent with previous studies regarding breast and prostate cancer (6,20). However, a significant association was not observed between WASF3 expression and lymph node metastasis in the present study. This may be due to the smaller sample size, which did not provide adequate power to detect such a difference. Furthermore, the present findings indicated a significant association between WASF3 expression and a reduced overall survival in the univariate and multivariate analyses. These results indicate that WASF3 may have prognostic value and may present as a possible therapeutic target for the treatment of lung cancer.

WASF3, as a member of the Wiskott–Aldrich syndrome protein/WAVE family of structurally and functionally associated proteins, is significant in the regulation of actin polymerization in the cytoskeleton, cell motility and cancer cell invasion (5,6,8,23–25). Additionally, WASF3 has been hypothesized to be involved in cancer invasion in numerous cancer cell lines (5,8–10,20,21,26,27), which implies that WASF3 may be critical in cancer progression and metastasis. Fernando *et al* (20) reported that the expression of WASF3 is stronger in prostate cancer tissues when compared with normal tissues and the expression of WASF3 was found to be significantly correlated with advanced human prostate cancer. The findings of the present study concerning NSCLC have been corroborated by similar findings in breast cancer patients; Kulkarni *et al* (21) observed that WASF3 expression was increased in the tumors of patients who developed distant metastases and markedly upregulated in the more aggressive triple-negative breast cancer patients. The data from the present study indicates that WASF3 may present as a useful biomarker for cancer progression and metastasis.

A significant correlation between the expression levels of WASF3 and the stage of lung cancer was observed in the present study. Furthermore, the patients exhibiting WASF3-positive expression were associated with a poorer prognosis when compared with those exhibiting WASF3-negative expression. Notably, the protein expression of WASF3 was not significantly increased in patients with lymph node metastases when compared with those without lymph node metastases. This was not consistent with previous results regarding breast cancer (21); however, a larger sample size may be required in future study. Zhang *et al* (22) found that WASF3 was overexpressed in colorectal cancer tissues, however, the colorectal cancer patients with WASF3 expression were associated with a good prognosis. The findings of the present study conflict with the abovementioned results regarding colorectal cancer. An explanation for this may be that there are numerous complex factors, which affect the interactions that occur *in vivo*. For example, the p38 mitogen-activated protein kinase (MAPK) signaling pathways, which are activated by WASF3, may possess a selective advantage in several types of cancer (28). The activated p38 MAPK signaling pathway may act as either a tumor suppressor (29,30) or as a tumor promoter depending on the type of cancer (31–33). Therefore, further investigation into the underlying mechanisms are required.

In conclusion, the present study identified that WASF3 was upregulated in NSCLC tissues, which indicates a potential role for WASF3 in the pathogenesis of NSCLC. Notably, this result provides clear support with regard to the function of WASF3 as a metastasis promoter protein. Furthermore, it was found that WASF3 may serve as a predictive marker of overall survival in NSCLC patients and may provide a potential target for anti-tumor therapy. However, in order to fully elucidate the exact function of WASF3 in NSCLC, further, larger studies are required.

## Figures and Tables

**Table I:** Characteristics of the non-small cell lung cancer patients (n=134).

Variable	qPCR analysis (n=38)	IHC analysis (n=96)
Gender		
Female	23	56
Male	15	40
Age, years		
<60	22	44
≥60	16	52
Histological subtype		
Adenocarcinoma	20	52
Squamous cell carcinoma	18	44
Differentiation status		
Well	14	30
Moderate	12	42
Poor	12	24
Lymph node metastasis		
Negative	16	42
Positive	22	54
Tumor staging		
IA–IB	16	32
IIA–IIB	16	44
IIIA	6	20

qPCR, quantitative polymerase chain reaction; IHC, immunohistochemistry.

**Table II:** Correlation between WASF3 mRNA expression and the clinicopathological features.

		WASF3 expression	
		
Variable	Patients, n	Cancer tissue^a^	P-value
Histological subtype			0.010
Adenocarcinoma	20	5.5852±0.4402	
Squamous cell carcinoma	18	4.0062±0.3686	
Lymph node metastasis			0.815
Negative	16	4.9254±0.5049	
Positive	22	4.7732±0.4094	
Differentiation status			0.241
Well	14	4.1408±0.5059	
Moderate	12	5.1874±0.6482	
Poor	12	5.2997±0.4434	
Tumor staging			0.013
IA–IB	16	4.1336±0.3875	
IIA–IIB	16	4.8171±0.4960	
IIIA	6	6.7677±0.6696	

a Data are presented as the mean ± standard error of the mean.

WASF3, Wiskott-Aldrich syndrome protein family member 3.

**Table III:** Association between WASF3 protein expression and clinicopathological parameters in non-small cell lung cancer.

		WASF3 expression		
			
Variable	Patients (n)	Pos. (n=53)	Neg. (n=43)	P-value
Gender				0.274
Male	56	31	25	
Female	40	22	18	
Age, years				0.543
<60	44	24	20	
≥60	52	29	23	
Histological subtype				0.001
Adenocarcinoma	52	37	15	
Squamous cell carcinoma	44	16	28	
Differentiation status				0.481
Well	30	14	16	
Moderate	42	24	18	
Poor	24	15	9	
Lymph node metastasis				0.623
Negative	42	22	20	
Positive	54	31	23	
Tumor staging				0.024
IA–IB	32	12	20	
IIA–IIB	44	26	18	
IIIA	20	15	5	

WASF3, Wiskott-Aldrich syndrome protein family member 3; Pos., positive; Neg., negative.

**Table IV:** Cox proportional hazard regression model analysis.

	Univariate analysis			Multivariate analysis		
		
Variable	Relative risk	95% CI	P-value	Relative risk	95% CI	P-value
Gender						
Male vs. Female	1.218	0.739–2.007	0.440	1.400	0.842–2.327	0.194
Age, years						
<60 vs. ≥60	0.724	0.443–1.185	0.199	0.642	0.383–1.075	0.092
Histological subtype						
AD vs. SCC	1.070	0.657–1.744	0.785	0.823	0.483–1.405	0.476
Differentiation status						
Well + Moderate vs. Poor	0.949	0.546–1.650	0.852	1.090	0.606–1.963	0.773
Tumor stage						
I+II vs. III	0.446	0.255–0.780	0.005	0.420	0.232–0.762	0.004
Lymph node metastasis						
Negative vs. Positive	0.740	0.452–1.213	0.233	0.891	0.527–1.507	0.667
WASF3 expression						
Negative vs. Positive	0.491	0.294–0.819	0.006	0.463	0.271–0.792	0.005

CI, confidence interval; AD, adenocarcinoma; SCC, squamous cell carcinoma; WASF3, Wiskott-Aldrich syndrome protein family member 3.
